# Complications of cochlear implants with MRI scans in different body regions: type, frequency and impact

**DOI:** 10.1186/s13244-022-01353-x

**Published:** 2023-01-16

**Authors:** Nilüfer Deniz Alberalar, Jonas Reis, Paula Louise Piechotta, Nick Lasse Beetz, Uli Fehrenbach, Dominik Geisel, Andreas Thomas, Harald Busse, Timm Denecke

**Affiliations:** 1 Department of Diagnostic and Interventional Radiology, Leipzig University Hospital, Leipzig, Germany; 2 Department of Radiology, Charité–University Medicine Berlin, Berlin, Germany; 3 Institute of Neuroradiology, LMU University Hospital, Ludwig Maximilian University of Munich, Munich, Germany

**Keywords:** MRI, Cochlea, Implant, MR complication, MR safety

## Abstract

**Objectives:**

The aim was to assess the type, frequency and impact of MRI-related complications in patients with cochlear implants (CI) and MRI indications in different body regions.

**Methods:**

For that purpose, the institutional radiology database of a single tertiary hospital was searched for patients with a CI who underwent MRI between 2001 and 2018. The number of MRI examinations and complications were retrieved from the patient record. Examinations were categorized into five distinct body regions or combinations thereof. Records of CI artifacts in the head also included basic information on diagnostic image quality.

**Results:**

Out of 1017 MRI database entries (examinations) of patients with a CI, 91 records were after implantation (71 patients) and 66 were attempted (no contraindications, 49 patients). In four cases (4/66, 6.1%), the magnet was dislocated and had to be replaced surgically. Three out of four severe complications occurred for examination regions outside the head*.* Thirteen MRI examinations were aborted due to pain (19.7%) and one because of artifacts—resulting in 48 scans (72.7%) completed successfully (36 patients). All cranial scans featured device artifacts in all sequences, but the majority of them did not affect proper imaging diagnostics in the respective region.

**Conclusion:**

This retrospective, single-center analysis of patients with MRI-conditional cochlear implants shows that MRI-related complications were common, at least in models with a fixed magnet, despite appropriate precautions and compliance with the manufacturers’ guidelines. MRI examinations of CI patients should therefore be indicated strictly until the exact causes have been clarified*.*

## Key points

1. Technical and procedural compliance with CI guidelines does not necessarily ensure safe examination.
2. High proportion of severe and mild complications for non-cranial examinations.
3. Need for extended informed consent for patients with CI.
4. Indications for MRI examinations with CI should still be handled with care.

## Background

Over the last years, cochlear implants (CI) have been increasingly used for the treatment of patients of all ages with severe sensorineural hearing loss [1–3]. The worldwide implementation of screening programs for newborns will effectively shift the time of diagnosis and of CI application to younger ages [4]. At the same time, the number of implantations will also increase in elderly patients considering a broader spectrum of indications and a higher life expectancy [2, 5]. In 2019, the United States National Institute on Deafness and Other Communication Disorders has estimated a number of nearly 740,000 registered devices implanted worldwide [6]. For the United States alone, this amounted to 118,000 and 65,000 devices for adults and children, respectively.

With rising numbers of MR scans and CI implantations per year, radiologists are confronted with an increasing number of diagnostic and therapeutic procedures in CI patients of all ages [2, 3, 7, 8]. Inpatient utilization of MRI procedures in Germany went from about 1.0 million per year in 2005 to about 1.5 millions in 2010 and has reached nearly 1.9 millions in 2020 [9]*.* Magnetic resonance imaging (MRI) is generally characterized by an excellent soft-tissue contrast and lack of ionizing radiation. It sees a growing use for many clinical questions but has strict limitations for devices with electrical or magnetic components. Following extensive experimental testing, each manufacturer issues a specific guideline on how to perform a corresponding MRI procedure safely [10]. Considering the differences between experimental and clinical settings, it is therefore crucial to also report and categorize the actual complications seen for routine MRI examinations.

The aim of this work is to assess the type and frequency of MRI-related complications in patients with cochlear implants at a single academic institution, a tertiary care hospital.

## Methods

This study was approved by the institutional review board of Charité—University Medicine Berlin, Germany (Reference EA1/110/18). The institutional radiological information system (RIS) was searched for variations of the (German) terms for cochlear implant and MRI examination between 2001 and 2018. The resulting MRI reports were reviewed manually to identify those cases where MRI had been performed with the implanted CI device. Matching data were available from three different campuses. Further exclusion criteria were missing specifications from the manufacturer, insufficient documentation of the CI model or declined patient consent.

A standard operating procedure (SOP) was followed for all patients: a radiology resident identified the specific safety requirements and scan protocol of the device manufacturer. Conditional MRI safety was then validated by a consultant radiologist. A radiologist also informed the patient about the general risks of an MRI examination and the specific risks related to a cochlear implant. These include symptoms like pain, nausea, dizziness or hypotension and technical issues like demagnetization or displacement of the inner magnet, dysfunction of the implant or displacement of the sensor, which all might necessitate revision surgery.

Patients were examined and prepared for MRI by the respective Otorhinolaryngology (ENT) or Radiology Department. This involved removal of the speech processor, transmitter coil and external magnet and attachment of a tight head bandage to keep the subcutaneous internal magnet in its silicone pocket. MRI was performed in two different 1.5 T models (Magnetom Avanto and Magnetom Aera, Siemens Healthcare, Erlangen, Germany). After MRI examination, the bandage was removed, and proper implant function was verified at the ENT department.

MRI complications were classified as mild or severe. A typical mild complication was pain experienced near the magnet or bandage, regardless of whether the MRI scan was successful, interrupted or aborted. Severe complications were those that caused device dysfunction or revision surgery, such as demagnetization or displacement of the inner magnet as well as displacement of the electrode or receive coil. The underlying body region was categorized as head–neck, spine, abdominopelvic, upper and lower extremity or combinations thereof. Cranial examinations also included an assessment of whether the artifacts prevented a proper interpretation of the imaging findings.

## Results

**Fig. 1:**
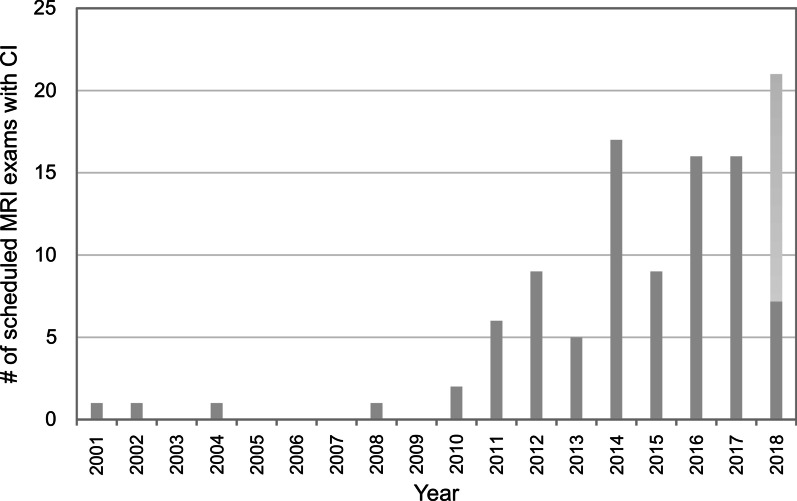
Bar graph showing the number of scheduled MRI examinations with cochlea implant per year between 2001 and 2018 at a single tertiary center (three campuses). The stacked bar for 2018 shows both the actual number for the first 4 months (7, dark gray) and the extrapolated value (21, light gray) for the entire year

The database search (2001–2018) resulted in 1017 records of CI patients with an MRI indication. A manual review of these records revealed 91 MRI examinations that were scheduled *after* CI implantation: 71 patients, 40 female (56.3%), 7–86 years old, mean age 51.3 ± (SD) 20.7 years. Over the first 9 years (and three campuses), however, only 4 patients were selected for MRI after CI implantation. From 2010 on, there has been a marked increase in selections with examinations per year typically reaching values above 15 between 2014 and 2018 (Fig. 1). In 7 underage patients, informed consent was given by the respective legal guardian. In 24 cases, required information was missing (informed patient consent or device documentation), and one examination was refused by the patient—leaving 66 MRI procedures attempted with a CI device. This corresponded to 49 patients (27 female, 54%) who had been examined at least once (41 patients once, 6 twice, 1 thrice, 1 ten times) for various indications.

Full documentation on the device was only given for 37 of these 66 MRI procedures, other reports did not specify the exact model number or series name and lacked any information about the implanted device. For the documented cases, the manufacturer was either Cochlear (31/37, about 84%) or Med-EL (6/37, about 16%). The most commonly used models were Cochlear Nucleus CI512 (19 times, 79% finished MRI examinations) and Cochlear Nucleus Freedom (7x, 86%)*.* The included patients were between 7 and 80 years old and had a mean age of 49.1 years (± SD of 20.2 years).

**Fig. 2:**
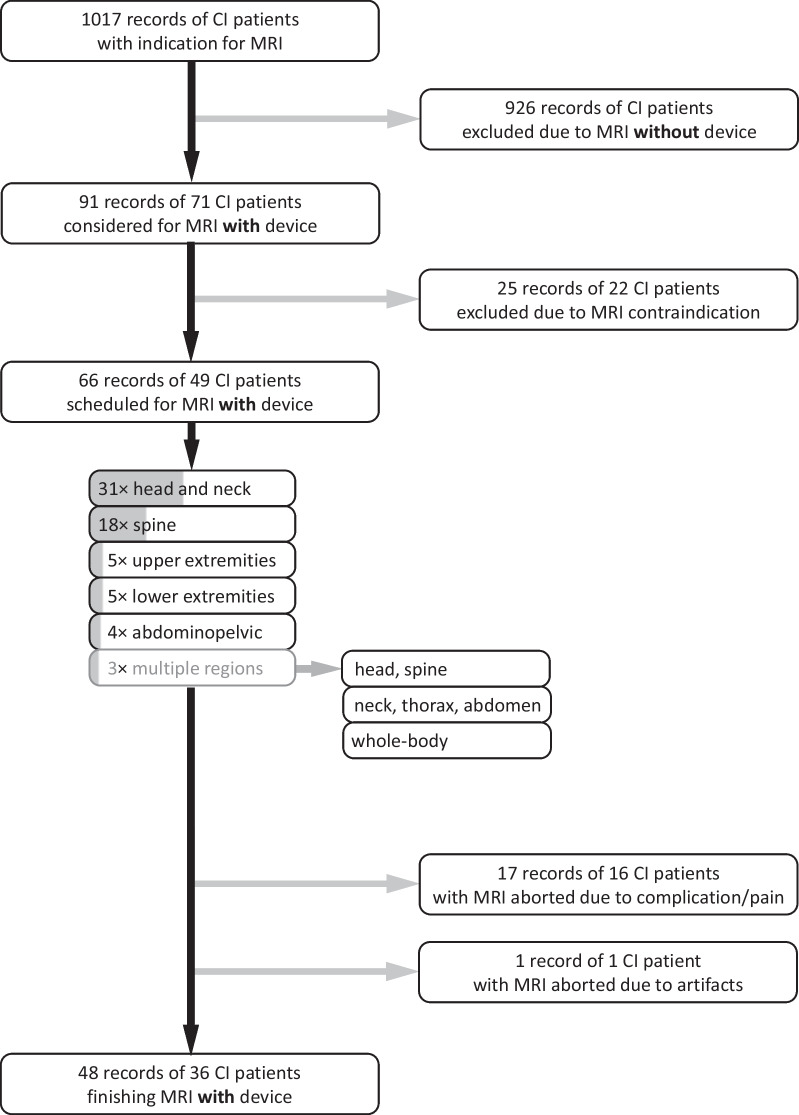
Flowchart of patients with a cochlear implant (CI) and MRI examinations between 2001 and 2018 from a single tertiary center and breakdown into body regions for scans *with* the CI device

**Fig. 3:**
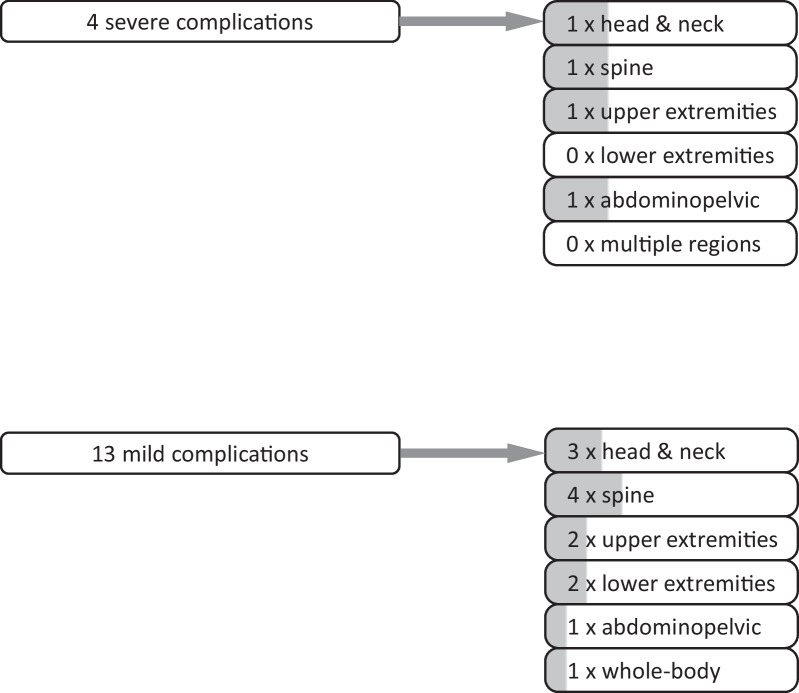
Number of severe and mild MRI complications observed for 66 MRI scans of CI patients with an MR-conditional device

Overall, one MRI was aborted due to artifacts (1/66, 1.5%) and 17 examinations were aborted with pain (17/66, 25.8%). In one of these terminations, the pain was related to the compression bandage. In four of the pain-related terminations (4/66, 6.1%), the subcutaneous (inner) magnet had become dislocated and required revision surgery (severe complication). The remaining 13 patients aborting MRI examination with pain were considered as mild complication. The remaining 48 examinations (72.7%) were completed successfully (36 patients), in 47/48 cases without pain and in one case with the patient tolerating the pain. Figure 2 gives an overview of the overall analysis. Figure 3 provides the anatomical details of the severe and mild complications recorded during the 66 post-implantation MRI scans. Dysfunction by displacement or damage to the receiver coil or electrode were not reported, meaning that no device had to replaced.

The breakdown into body location (Fig. 2) shows a dominance of head and neck (number: 31) and the spine (18) with fewer numbers in the upper (5) or lower extremities (5) and in the abdominopelvic region (4). Two patients had a combination of body regions, head and spine in one case, and neck, thorax and abdomen in the other, and a third patient had a whole-body MRI. Head and neck cases showed one severe (3.2% of 31, relative to number of examinations in respective region) and three mild (9.7% of 31) complications, while the spine had one severe (5.6% of 18) and four mild complications (22.2% of 18). The number of severe/mild complications for the remaining cases (percentages in parentheses, respectively) was 1/1 (25%/25% of 4) for the abdomen, 1/2 (20%/40% of 5) for the upper and 0/2 (0%/40% of 5) for the lower limb. The whole-body MRI ended with a mild complication (0%/100%), while the other combined scans were completed without any problems.

In 3 of 8 patients with multiple MRI examinations scheduled, technical success was inconsistent across different dates. After successful completion of a spine examination of a 25-year-old female patient, a follow-up abdominal scan had to be stopped due to pain and dizziness. About four months later, another attempt of the abdominal scan even resulted in a dislocation. A 62-year-old male patient had a mild complication (pain) upon first attempt of a head MRI, but finished without pain about 8 months later. A 53-year-old female required an MRI of the spine but experienced a severe complication with magnet displacement and pain. Ten days later, the spine MRI could be completed without problems after the inner magnet had been removed surgically.

Severe complications had occurred twice with a Cochlear Nucleus CI522 and once with a Nucleus CI512. For the fourth case, information about the exact model was retrospectively not available. One female CI patient had an anaplastic oligodendroglioma, a tumor with rather rapid progression that should be controlled more often (by MRI). Over a period of 3.5 years (age 35 to 39) and throughout the course of several neurosurgical interventions, she underwent a total of 10 cranial MRI scans with a Nucleus CI512 device without any complication or pain*. *

**Fig. 4:**
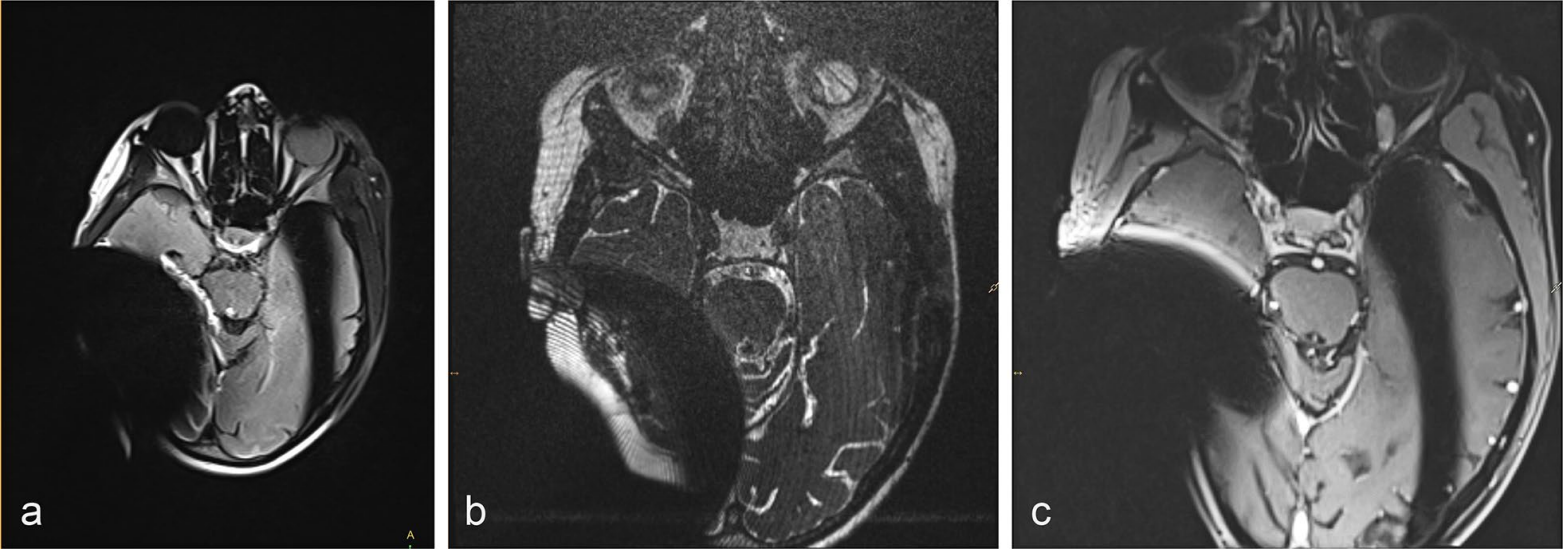
Examples of transverse gradient-echo (GRE) MR images illustrating extent of CI artifact in a 53-year-old female patient to depict cochlear anatomy and vestibulocochlear nerve on the contralateral side **a**: T2-weighted FLAIR image (FOV 250 × 203 mm^2^, slice thickness ST 3.0 mm, slice gap 0.6 mm), **b** T2/T1-weighted 3D CISS image, coherent balanced GRE using dual-excitation (FOV 180 × 180 mm^2^, ST 0.6 mm), **c** contrast-enhanced T1-weighted fat-saturated VIBE image, spoiled 3D GRE with volume interpolation (FOV 160 × 180 mm^2^, ST 1.0 mm)

All cranial MR images showed artifacts related to the inner magnet, and an example is shown in Fig. 4. Artifacts were rated as severe—preventing proper MRI diagnostics in the affected region—in 36.0% (9/25) of the reported cases (2 cases undocumented).

## Discussion

Our study covers a relatively large time span (2001–2018), but the number of cases before 2010 is low. Around that year, there is a clear increase in the number of recorded implantations although the cases per year still vary from year to year. This trend is in line with the global evolution of an increasing number of MRI examinations as well as CI implantations, which clearly calls for evidence-based measures to ensure patient safety, preserve image quality and limit costs. Our retrospective study has analyzed a relatively large number of CI patients with an MRI scan [11–14]. In the light of a worldwide growing number of cochlear implants, radiologists need to handle these patients in a safe manner [15, 16], which requires a thorough assessment of the individual risks in the first place.

Despite device preparation and strict scanning guidelines—regarding, for instance, specific absorption rate, main or spatial gradient field—a risk for severe complications remains. The ones seen here, all displacements of the inner magnet from its silicone pocket, demanded invasive surgical measures with extra risks and costs. They occurred although all general (staff training) and specific guidelines (see above) had been followed. This is in line with previous reports from an implant center where CI devices were dislocated despite adherence to the respective manufacturer’s recommendations [15–17]—and had to be relocated surgically.

Some sites have routinely performed a Stenvers view radiograph after MRI examination to rule out a magnet dislocation [17–19]. Such a procedure was not implemented here because magnet dysfunction or displacements were routinely checked in the ENT department—also sparing the patient from ionizing radiation. A compression bandage or cover is generally advised for most CI models to reduce the chances for magnet dislocation [11, 12, 16, 20]. In rare cases, however, the bandage itself may cause pain, as seen for one of our patients who then refused imaging.

In the prospective study by Pross et al., 5 of 42 MRI examinations (about 12%) were stopped early due to pain or anxiety [21]. Loth et al. have retrospectively also identified pain as the main reason for aborting a scan and the respective frequency was even higher (about 37%, 34/91 patients) [22]. An intermediate percentage for pain-related termination was observed in the present study (25.8%). The patient should therefore be clearly informed that MRI with a CI may cause pain in a way that the examination needs to be aborted. The above literature and own results show some variability on the order of tens of percent. This may be the result of the specific CI models implanted considering that some models have been reported to cause complications in different studies. For example, Leinung et al. have described CI dislocations for different models from Cochlear (5 × CI512 and 2 × CI24RE and 1 × CI532, two of them present here) and one model from Advanced Bionics (1 × HiRes 90 k) [15]. Four of these 9 patients had also reported pain (2 × CI24 RE and 2 × CI512). The same authors also mentioned that three of these dislocations occurred in (external) radiological centers without proper experience with CI patients—supposedly lacking extra care. Another factor for variability in CI complications across studies may be related to the practice of patient referral. Loth et al. have analyzed the questionnaires of CI patients and observed that only seven out of 55 (13%) radiology departments or practices that performed the MRI belonged to a center that also provided hearing implant surgery [22].

CI models with optimized magnet configuration may reduce the rate of complications. One approach (Synchrony, Med-EL) features a rotatable, self-aligning magnet to minimize torque [23]. According to the manufacturer, that device works up to 3 T and may be used without a head bandage. Eerkens et al. have experimentally measured forces and torques of six CI models at critical locations of an MRI system (1.5 T) [24]. Two CI devices showed magnet dislocations (Cochlear Nucleus CI24RE and Advanced Bionics HiRes Ultra) caused by torque inside the magnet bore, while two other models (Med-EL Concerto and Synchrony) apparently remained in place. These Med-EL findings are in line with our six confirmed applications, although one minor complication was reported for a Concerto device*.*

A clear recommendation for or against a specific CI model is difficult here, because our analysis may be biased by the incomplete CI recordings. Even with upcoming, potentially safer CI models, radiologists will still need to decide for patients with older devices. It is generally important to stay up to date on device guidelines, especially because they may differ between countries, where specific changes may come into effect at different time points.

The two main causes for magnet dislocation are translational and rotational forces. Translational forces are highest near the bore entry along the system’s inner wall but rapidly drop toward the isocenter, practically vanishing for much of the system’s interior (bore). This is in contrast to the rotational forces (torque), which are highest practically anywhere inside the bore. The exact effect also involves the angle between the magnetic fields of the MRI system and the CI magnet. For a given patient, the orientation of the CI magnetic field will depend on CI design, point of implantation (head curvature) and, ultimately, the pose of the head. Although Vincent et al. have investigated an older CI model, their report from 2008 provides further details [25]. Specific recommendations for patient pose and entry into the MRI system have also been described [23]. 

Replacement of the magnet is usually a minor surgical procedure under local anesthesia but still carries a small risk of local infection [15, 17], which should be part of informed consent.

Pross et al. could previously not identify any specific body part where pain occurred preferentially [21]. This finding is confirmed by our results with mild/severe complications seen for MRI examinations in different parts of the body.

Our case of a female patient with a total of 10 cranial MRI scans with the device (without any complication or pain) obviously introduces a bias for all percentages (for example, complication rate) reported relative to the number of individual examinations instead of patients. Other patients with multiple examinations, however, showed clear differences in procedural outcome, in particular one patient with two failed MRI scans (mild and severe complication) despite a successful initial examination with cochlear implant, albeit in a different body region. This highlights the importance of strictly verifying the clinical indication for any MRI examination.

Patients undergoing cranial MRI should also consent to the fact that the diagnostic image quality may be limited by artifacts, in particular, ipsilateral to the CI [26]. Cranial artifacts were not analyzed in more detail due to the incomplete reporting of CI models—also, previous studies have addressed this issue already. Todt et al., for example, have observed no significant differences between models in the diameter and severity of artifacts, also for different sequences [27]. Another study has compared the artifacts of a single model (Nucleus CI512) at different head positions. The authors showed that a high-resolution 3D T2w DRIVE sequence created significant artifacts of the temporal structures on the ipsilateral side, unlike a high-resolution TSE 2D T2w sequence [1]. 

In an effort to reduce the image artifacts caused by metal implants, pulse sequences like SEMAC (slice encoding for metal artifact correction) have been designed in the late 2000s [28]. Amin et al. have recently used a view angle tilting technique to substantially reduce the signal void and improve the visibility of the posterior fossa of CI patients [29]. 

Severe complications seem to mostly occur in small radiological centers without proper CI expertise, and CI patients are often not undergoing MRI at the same center where the CI was implanted [22, 29].

**Fig. 5:**
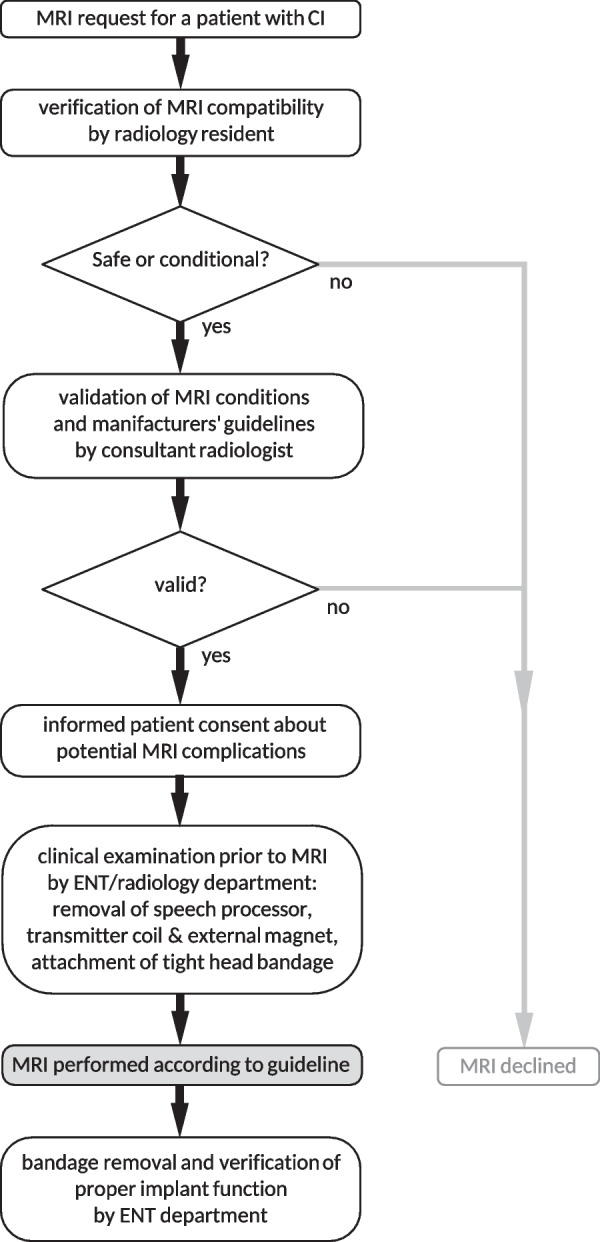
SOP diagram for MRI examinations with cochlear implants

We believe that an SOP for MRI examinations with cochlear implants (Fig. 5) is a simple and highly effective measure to minimize the risks for patients and treating staff. Responsibility for preparation and examination should be shared among ENT doctors, CI technicians, radiologists and radiology technicians. Technicians should be trained regularly in handling a particular implant and compression bandage. Radiologists should also inform the patient explicitly before CI deactivation and bandage application. Given the extra risks and medical liability issues, it is advisable to provide ample information and documentation.

There are several limitations in this study. The main ones are the retrospective design based on radiological reports rather than questionnaires, and the overall incomplete documentation. We still believe that our results serve as a valuable practical reference as they were obtained at different campuses, for several models and over the course of more than a decade.

Over the next years, the challenge is likely to persist, considering that the number of CI implantations and MRI examinations is still growing. CI devices with improved magnet designs will certainly contribute to less (severe) complications and overall benefit for the patient in the long run. From a practical viewpoint, however, clinicians and radiologists will still need to know about the handling of less advanced (older) models to ultimately ensure patient safety. Therefore, the individual MRI indication should be verified strictly for all CI patients.

## Conclusion

Despite appropriate precautions and compliance with the manufacturers’ guidelines, MRI examinations with a cochlear implant, at least in models with a fixed magnet, still have a considerable rate of complications. Indications for MRI should therefore be handled more strictly and accompanied by an extended informed consent reflecting the latest knowledge about the various factors, such as MRI model, CI model, body part and patient entry.

## Data Availability

The datasets used and/or analyzed during the current study are available from the corresponding author on reasonable request.

## Abbreviations

CI: Cochlear implant
ENT: Ear nose throat/otorhinolaryngology
MRI: Magnetic resonance imaging
RIS: Radiological information system
SOP: Standard operating procedure
